# Advances in Non-Invasive Screening Methods for Gastrointestinal Cancers: How Continued Innovation Has Revolutionized Early Cancer Detection

**DOI:** 10.3390/cancers17071085

**Published:** 2025-03-24

**Authors:** Dushyant Singh Dahiya, Sheza Malik, Ruchir Paladiya, Sidra Ahsan, Haniya Wasim, Hareesha Rishab Bharadwaj, Abhishek Goel, Ali Jaan, Umar Hayat, Fariha Hasan, Sneh Sonaiya, Hassam Ali

**Affiliations:** 1 Division of Gastroenterology, Hepatology & Motility, The University of Kansas School of Medicine, 3901 Rainbow Boulevard, Kansas City, KS 66160, USA; 2 Department of Internal Medicine, Rochester General Hospital, Rochester, NY 14621, USA; 3 Department of Internal Medicine, University of Connecticut Health Center, Farmington, CT 06269, USA; 4 Department of Internal Medicine, Ochsner LSU Health—Fairfield Medical Office Building, 1801 Fairfield Ave, Shreveport, LA 71101, USA; 5 Department of Internal Medicine, AdventHealth West Florida, Altamonte Springs, FL 32701, USA; 6 Faculty of Biology Medicine and Health, The University of Manchester, Manchester M13 9PL, UK; 7 Department of Internal Medicine, Cape Fear Valley Medical Center, Fayetteville, NC 23804, USA; 8 Department of Internal Medicine, Geisinger Wyoming Valley Medical Center, Wilkes-Barre, PA 18711, USA; 9 Department of Internal Medicine, Cooper University Hospital, Camden, NJ 08103, USA; 10 Department of Internal Medicine, University of Nevada Las Vegas, Las Vegas, NV 89154, USA; 11 Division of Gastroenterology, Hepatology & Nutrition, Brody School of Medicine, East Carolina University, Greenville, NC 27858, USA

**Keywords:** gastrointestinal cancers, early detection, non-invasive screening, artificial intelligence, challenges

## Abstract

Early detection is important in reducing mortality from gastrointestinal (GI) cancers, but conventional screening methods can be invasive and expensive. Non-invasive diagnostic approaches hold promise for more efficient surveillance. For gastric cancer, oral rinse tests assessing the oral microbiome and circulating tumor DNA (ctDNA) analysis have shown improved specificity, while stool-based assays and FDA-approved blood-based tests such as Shield are revolutionizing colorectal cancer screening. Pancreatic cancer detection benefits from liquid biopsy technologies targeting KRAS mutations, exosomal markers, and VOC breath analysis. Hepatocellular carcinoma (HCC) surveillance is evolving with ctDNA methylation panels plus AI-driven radiological assessments. These innovations address long-standing challenges in early GI cancer diagnosis by increasing sensitivity and patient comfort. This review highlights the most recent advances in non-invasive GI cancer screening, offering a hopeful future for early detection and paving the way for personalized interventions.

## Simple Summary

This review explores how recent innovations in non-invasive screening methods have transformed the early detection of gastrointestinal cancers, which include esophageal, gastric, colorectal, pancreatic, liver, and small bowel cancers. Traditional screening techniques, while effective, are often invasive, expensive, and uncomfortable, leading to low participation rates, especially in populations without symptoms or with limited healthcare access. This paper highlights new, less invasive methods such as blood tests that detect genetic material from tumors, stool-based DNA tests, breath analyses for chemical markers, and advanced imaging techniques assisted by artificial intelligence. These approaches improve patient comfort, boost participation, and detect cancer earlier, when treatment is more likely to succeed. Despite these advancements, challenges such as test accuracy, cost, accessibility, and regulatory approvals remain. Addressing these issues will require collaboration among researchers, healthcare providers, and policymakers. The continued development and adoption of these non-invasive techniques could significantly reduce deaths from gastrointestinal cancers by enabling earlier diagnosis, personalized treatment, and better health outcomes for patients worldwide.

## 1. Introduction

**Figure 1:**
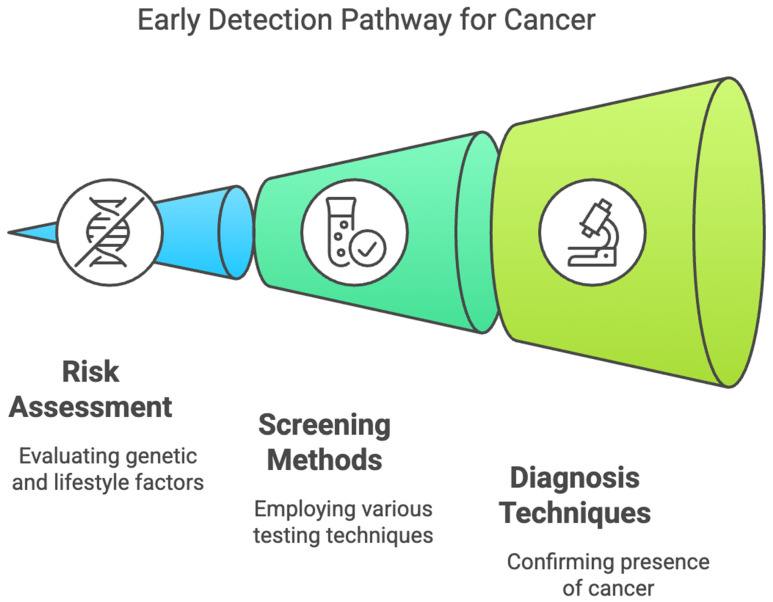
Early Detection Pathway for Gastrointestinal Cancers.

The early diagnosis of gastrointestinal cancers is essential for better survival and to reduce the burden of malignancies worldwide. However, screening methods such as endoscopy, colonoscopy, and imaging techniques, though effective, are usually invasive, expensive, and resource-consuming. These limitations, in turn, lead to low adherence, particularly in asymptomatic patients or in resource-poor settings, thus leading to late diagnosis and a poor prognosis. There is an increasing need for non-invasive or minimally invasive approaches that will improve early detection, patient compliance, and access to screening programs. Over the last years, technological development in cancer screening for GI malignancies has shifted toward the use of molecular biomarkers, AI-assisted imaging, and novel metabolic tests for more superficial stages [1,2,3,4,5,6,7,8,9,10]. Liquid biopsies, investigating circulating tumor DNA, miRNAs, exosomal markers, and other components that originate from tumor cells, have gained promising results in detecting gastrointestinal carcinomas. As promising as these times may seem, there are many issues to be overcome before non-invasive screening methods see wide acceptance in clinical use. Among these, the main issues to be considered are variabilities in sensitivity and specificity, large-scale validation studies, regulatory challenges, cost-effectiveness, and equity in health. Standardization of the biomarker panel across diverse populations is a critical step toward consistency in diagnostic precision. In addition, although AI-based diagnostics have very bright prospects, strong computational infrastructures, regulatory approvals, and clinician training will be required. Implementation will also be complicated by ethical considerations regarding genetic testing, data privacy, and incidental findings. Furthermore, thoughtful policy development and patient education will also be required. This review reflects on the recent progress that has been made for non-invasive screening of GI cancers, mainly with respect to their potential game-changing role for early cancer detection and the challenges still to be overcome on the path toward their broad clinical translation (Figure 1).

## 2. Esophageal Cancer

**Figure 2:**
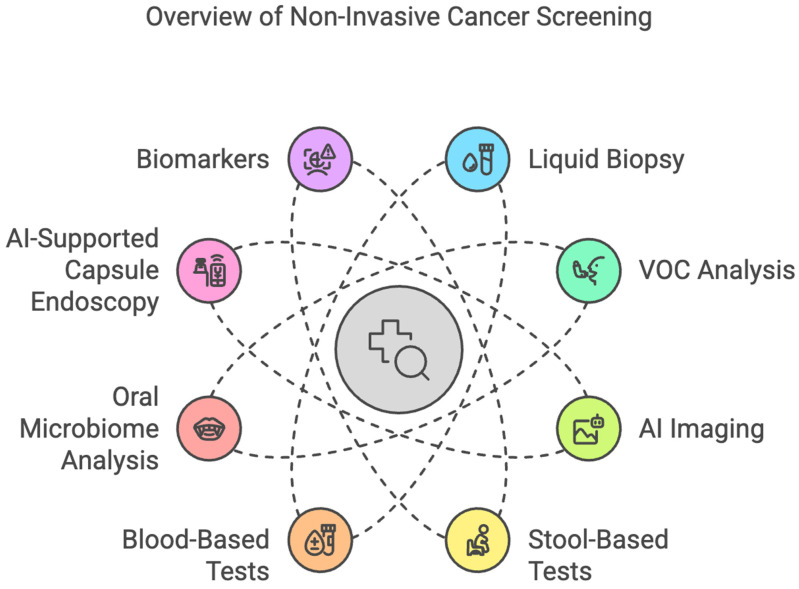
Overview of Non-Invasive Cancer Screening Methods.

Esophageal cancer (EC) is the sixth most common cause of cancer-related death worldwide [1,2]. The incidence of esophageal squamous-cell carcinoma (ESCC) has reduced in most parts of the world due to decreases in cigarette smoking and heavy alcohol consumption [3,4]. However, due to the rising prevalence of gastroesophageal reflux disease, Barrett’s esophagus, and obesity, the annual incidence rate of esophageal adenocarcinoma (EAC) has risen by 7–8% in the United States and will constitute 75% of all esophageal cancer cases by 2030 [5]. The poor survival rate of 15–25%, despite advances in treatment, points towards delayed presentation in most cases [6]. There are various treatment options, such as endoscopic mucosal resection (EMR) and surgical intervention, that can be performed for early-stage esophageal cancer. Although invasive endoscopic surveillance screening is indicated in certain high-risk populations, various non-invasive tests have been developed for the early detection of EC screening (Figure 2).

### 2.1. Liquid Biopsy

A liquid biopsy refers to a blood test that identifies circulating abnormal cells or their components, such as DNA or microRNA (miRNA), to detect the precursors or presence of diseases, mostly cancers.

#### 2.1.1. Blood Autoantibodies

Autoantibodies have emerged as potential biomarkers for the early detection and prognosis of esophageal cancer (EC), particularly esophageal adenocarcinoma (EAC) and esophageal squamous-cell carcinoma (ESCC). These autoantibodies are produced by the immune system in response to tumor-associated antigens and can be detected in serum. A meta-analysis of 55 studies (43 serum-based and 12 plasma-based) reported 91% specificity for the detection of squamous dysplasia and ESCC but a sensitivity of only about 45%, highlighting their utility as an adjunctive diagnostic tool. In comparison, circulating tumor DNA (ctDNA) provides additional insights into tumor dynamics [7,8]. A previous study demonstrated that ctDNA was 90% positive in locally advanced tumors, nodal involvement, or metastatic disease and accurately predicted disease-free survival intervals compared to imaging modalities such as PET and CT scans [9]. Combined with autoantibody analysis, this may enhance diagnostic accuracy, as tumor protein 53 (TP53) mutations detected through ctDNA are associated with a negative prognostic value in EAC [10]. Nevertheless, these remain a cost-effective, minimally invasive option with the potential for integration into broader screening protocols.

#### 2.1.2. Circulating MiRNAs

miRNAs are small non-coding RNAs that bind to target messenger RNAs and degrade or inhibit them, and can be easily detected in the serum owing to their abundant expression. A pooled analysis of 27 studies reported an 80% sensitivity and 81% specificity for ESCC [11]. Similar results were observed in a meta-analysis of 69 studies (57 studies with single miRNAs and 12 studies with multiple miRNAs) that showed 77% sensitivity and 78% specificity [8]. Thus, the concept of miRNAs holds potential as a non-invasive screening tool, and further studies are underway.

#### 2.1.3. Methylated DNA Markers (MDMs)

MDMs have been demonstrated to be informative markers of neoplasia, which occurs via two fundamental changes: hypermethylation of CpG islands, leading to the silencing of tumor suppressor genes, or hypomethylation of repetitive genetic elements, leading to oncogenic activation. A recently published study on non-endoscopic balloons from 86 individuals reported 90% sensitivity and 92% specificity for CCNA1, and VIM DNA methylation detected BE metaplasia [12]. The five-MDM panel was able to detect 74% of esophageal cancers (74% of EAC and 78% of ESCC), with an overall AUC of 0.93 [13]. The study included eighty-five cases (seventy-six EAC and nine ESCC) and ninety-eight controls. Active research in this area can help transform EC screening in resource-poor endemic areas.

#### 2.1.4. Circulating Tumor Cells (CTCs)

CTCs have been studied for their prognostic utility in esophageal cancer. Their presence typically demonstrates a strong correlation with nodal metastases and poor progression-free survival in patients with esophageal cancer. A threshold of two CTCs per 3.2 mL of peripheral blood reported 70.54% sensitivity and 96.74% specificity for differentiating EC patients from healthy controls using a negative enrichment–fluorescence hybridization (NE-FISH) technique among 129 patients who were newly diagnosed with EC, 17 with benign disease, and 75 healthy donors [14]. Another study with a similar objective reported 86.3% sensitivity and 90.3% specificity for two CTCs per 7.5 mL of blood using the fluid–aspiration separation technique among 73 patients [15].

### 2.2. Volatile Organic Compounds (VOCs)

VOCs are products of the human and gut bacterial metabolism that can be detected in breath. Tumor-induced metabolic changes alter VOC profiles and provide tissue metabolomic biomarkers for early detection. A Japanese single-center study reported significantly higher levels of acetonitrile, acetic acid, acetone, and 2-butanone in ESCC patients than in controls [16]. A promising role of VOC induction by methionine regulation has been reported in distinguishing EC cells from normal esophageal epithelial cells [17]. A multi-center validation study reported 86.2% sensitivity and 89.5% specificity with a five-VOC model to differentiate EC patients from healthy people [18]. Another serum-based five-VOC model with 55 EC patients and 84 healthy volunteers showed 94.1% sensitivity and 96% specificity, emphasizing the future diagnostic value of the test. A urinary model with six VOCs also differentiated gastroesophageal cancer patients from their non-cancerous counterparts with an AUC of 0.904 [19].

### 2.3. Esophageal Cytology Specimens Combined with Biomarkers

Non-endoscopic cell collection devices allow the collection of cells from the entire esophagus for biomarker analysis. Various sampling devices are available, including Cytosponge, which consists of a small, compressed sponge enclosed in a gelatin capsule attached to a string. Once swallowed, the capsule dissolves in the stomach, releasing the sponge. The sponge is then pulled back through the esophagus, collecting epithelial cells along its path for biomarker analysis (Medtronic). Another option is EsophaCap, which involves a collapsible sponge encased in a capsule that expands after ingestion. The expanded sponge collects a sample of esophageal cells as it is withdrawn through the esophagus (PAVmed). EsoCheck combines an inflatable balloon with a specialized retrieval system. After the balloon is inflated in the lower esophagus, it collects cells from the esophageal lining as it is withdrawn (PAVmed), allowing esophageal cytology collection for the analysis of BE- and EAC-associated biomarkers such as trefoil factor 3 (TFF3) and MDMs. The combination of the EsoCheck device and the EsoGuard assay demonstrated 85% overall sensitivity and 85% specificity for the detection of BE and EAC, with 100% sensitivity for cancer detection [20]. A pilot study using a combination of EsophaCap and two-MDM panels was found to have 100% sensitivity and specificity for BE detection [21].

### 2.4. Advances in Imaging

#### 2.4.1. Unsedated Transnasal Endoscopy (uTNE)

Unsedated Transnasal Endoscopy (uTNE) involves the use of an ultrathin endoscope (diameter < 6 mm), which is introduced through the nasal cavity without the need for intravenous sedation and can be performed in an office setting. A randomized control trial of 459 patients reported a higher participation rate than sedated esophagogastroduodenoscopy (sEGD), with shorter procedure and recovery times [22].

#### 2.4.2. Capsule-Based Imaging

Esophageal capsule endoscopy was performed for the direct visualization of the esophagus. A meta-analysis reported a sensitivity of 77% and a specificity of 86% for BE diagnosis [23]. The major limitation of this method is its inability to retrieve biopsy specimens. However, tethered capsule endomicroscopy can capture high-resolution cross-sectional images. Several preliminary studies have reported the role of this imaging modality in BE. A multi-center prospective study of 147 patients with biopsy-proven BE reported a shorter procedure time and ease of use compared to the standard technique [24].

### 2.5. Oral Microbiome Analysis

Several studies have reported the role of microbial dysbiosis in the oral cavity in esophageal carcinogenesis through chronic inflammation, nitrosamine production, and immune modulation. Lifestyle factors such as alcohol consumption, smoking, and betel nut use are major contributors to oral dysbiosis and EC development. An AUC of 0.87 was reported during analysis of 249 oral flora samples, which identified various markers, including Neisseria perflava and Haemophilus parainfluenzae, to distinguish ESCC from controls [25]. Another recent study also identified oral metagenomic classifiers that differentiated EC patients from controls, with an AUC of 0.791 [26]. Therefore, the incorporation of microbiome-focused approaches into existing therapeutic paradigms may enhance EC management, mitigate adverse effects, and improve patient outcomes.

## 3. Gastric Cancer

Recent research has identified multiple non-invasive biomarkers for the diagnosis and prognosis of gastric cancer (GC), the fifth most diagnosed cancer and the third leading cause of cancer-related deaths worldwide. Currently, there are no universal screening guidelines for GC. However, due to the rising incidence of gastric cancer and the increasing feasibility of non-invasive screening methods, these tests are being actively investigated.

### 3.1. Liquid-Based Tests

A nine-gene panel derived from genome-wide transcriptome expression profiling effectively distinguishes GC from non-cancerous tissues. Additionally, the altered expression of circulating microRNAs (miRNAs) has been implicated in gastric tumorigenesis, with miR-34b/c methylation emerging as a promising biomarker for assessing the risk of multiple GCs [27].

### 3.2. Circulating Tumor Cells

As a liquid biopsy, CTCs offer a minimally invasive method of monitoring tumor dynamics and treatment responses in GC. Compared to analyzing adjacent gastric tissue, which may exhibit aberrant gene signatures distinct from both tumor and healthy tissues, using circulating biomarkers is less labor-intensive. A meta-analysis reported that CTC detection methods for GC have a combined sensitivity of 42% and a specificity of 99%. Markers such as EpCAM and CD44 enhance diagnostic accuracy, with EpCAM++ CD44++ cell detection achieving 92.3% sensitivity and 100% specificity [28].

### 3.3. Circulating Tumor DNA

DNA methylation patterns, particularly in gastric mucosa and ctDNA, have shown diagnostic potential. CancerSEEK, a multianalyte blood test, achieves high specificity (>99%) and moderate sensitivity (70%) for GC by analyzing ctDNA mutations and protein biomarkers [29].

### 3.4. Exosomes and Non-Coding RNAs

Exosomes regulate and participate in various physiological and pathological processes. They can encapsulate non-coding RNAs—such as miRNAs, circular RNAs (circRNAs), and long non-coding RNAs (lncRNAs)—protecting them from degradation and facilitating intercellular communication [30]. Panels of plasma lncRNAs, including AOC4P, BANCR, and TINCR, as well as circRNAs such as hsa_circ_002059 and hsa_circ_0000190, can distinguish GC from non-GC tissues with high diagnostic accuracy. Furthermore, altered circRNA–miRNA–mRNA interactions underscore the role of these molecules in tumorigenesis and highlight their potential clinical utility in both diagnosis and prognosis [31,32].

### 3.5. Oral Rinse Tests

The oral microbiome shows substantial promise as a non-invasive diagnostic tool for gastric cancer. In one study involving 98 participants (62% female and 31% Hispanic), oral microbiome profiles were compared among three groups: those with gastric cancer, those with pre-malignant lesions, and controls. The analysis identified 32 bacterial genera that differed significantly between control and gastric cancer groups and 23 genera between control and pre-malignant groups. Helicobacter, Selenomonas, and Lactobacillus were more abundant in the gastric cancer and pre-malignant groups, while Parabacteroides, Alistipes, and Phascolarctobacterium were more prevalent in controls. Notably, the oral microbiome profiles of the pre-malignant and gastric cancer groups were highly similar, reinforcing its potential as an early detection strategy for GC [33].

### 3.6. VOC Breath Analysis

VOCs in exhaled breath offer a non-invasive method for the early detection of GC [34]. Markar et al. used SIFT-MS to profile VOCs in 72 GC patients, along with gastroesophageal junction and esophageal cancer cases, and 172 controls. Five distinct compounds were identified, resulting in a logistic regression model with an AUC of 0.85, 80% sensitivity, and 81% specificity. While promising, the model could not distinguish between gastric and esophageal cancers and was primarily applied to advanced disease stages, requiring further validation for early GC detection [35]. Studies have also shown elevated aldehydes, such as hexanoic acid, butanal, and decanal, in GC patients compared to controls. These compounds originate from lipid peroxidation caused by cancer-induced oxidative stress. Aldehyde detoxification, mediated by aldehyde dehydrogenase (ALDH), is impaired in GC, contributing to aldehyde accumulation. For example, ALDH3A2 downregulation has been linked to poor prognosis in esophagogastric cancers. Understanding VOC profiles, particularly aldehydes and carboxylic acids, may enhance GC diagnostic accuracy [36,37].

## 4. Colorectal Cancer

Colorectal cancer remains one of the leading causes of cancer-related mortality worldwide, with early detection playing an important role in improving survival rates [38]. While colonoscopy remains the gold standard for screening, there is growing interest in less invasive screening modalities (Table 1).

### 4.1. Blood-Based Tests

The U.S. Food and Drug Administration (FDA) has approved Epi proColon 2.0, a test that detects methylated SEPT9, a molecular biomarker shed into the bloodstream by colorectal cancer cells. It is indicated for adults aged 50 and older at average risk for colorectal cancer who have been offered but have not completed conventional screening through stool-based or direct visualization methods [48]. Another blood-based test, Shield, is available for adults aged 45 and older at average risk of colorectal cancer. Shield analyzes plasma DNA for specific alterations, including mutations associated with malignancy. A study by Chung et al. reported 83% sensitivity for colorectal cancer and 90% specificity for advanced neoplasia [39], outperforming traditional biomarkers such as SEPT9. Shield provides a convenient alternative for individuals reluctant to undergo colonoscopy or stool-based tests, potentially improving adherence and early detection. However, blood-based tests are not yet included in clinical guidelines as first-line screening options for colorectal cancer [49]. Advanced microfluidic platforms and negative enrichment techniques now enable highly specific circulating tumor cell (CTC) isolation, while single-cell RNA sequencing allows molecular characterization. Combining CTCs with exosomal RNA could enhance colorectal cancer screening and post-treatment monitoring [50,51].

### 4.2. Stool-Based Tests

Stool-based testing for colorectal cancer (CRC) primarily includes guaiac-based fecal occult blood testing (gFOBT) and fecal immunochemical testing (FIT), both of which detect blood in stool, a common marker of CRC. While FIT provides better sensitivity and fewer false positives than gFOBT, both tests have limitations in detecting early-stage cancers and adenomas [52]. In contrast, multi-target stool DNA testing (mt-sDNA) identifies genetic mutations and epigenetic alterations linked to CRC. The FDA-approved mt-sDNA test, Cologuard, combines FIT with DNA analysis to improve detection rates for CRC and advanced precancerous lesions [53]. The literature has demonstrated its superior sensitivity compared to FIT and gFOBT, with approximately 92% sensitivity for CRC detection, 42% for advanced adenomas, and 80% for all cancer stages combined [40]. Since it detects DNA alterations independent of bleeding, mt-sDNA increases the likelihood of identifying CRC, particularly in early-stage disease. However, it comes with high costs, limited availability, and the potential for false positives or negatives, necessitating colonoscopy confirmation for positive results [54]. Current recommendations suggest mt-sDNA screening every three years [55]. Fecal methylation testing represents a significant advancement in stool-based CRC screening by identifying aberrant DNA methylation patterns in shed colorectal epithelial cells. Methylation of genes such as SEPT9, SFRP2, NDRG4, and BMP3 has been strongly associated with CRC and advanced adenomas. A meta-analysis reported that fecal methylation biomarkers achieved sensitivity values of 75–90% and specificities over 85% for CRC detection, outperforming FIT in some populations [56]. As stool-based testing evolves, the combination of FIT, mt-sDNA, and fecal methylation assays could improve early detection and reduce CRC mortality by offering a non-invasive yet highly effective screening option for broader populations [57].

### 4.3. Computed Tomographic (CT) Colonography

Virtual colonoscopy, also known as computed tomographic (CT) colonography, is often utilized as well. This technique utilizes advanced X-ray imaging to generate detailed, computer-reconstructed images of the colon and rectum, allowing for the identification of polyps and other abnormalities. Like colonoscopy, virtual colonoscopy requires thorough bowel preparation but is significantly less invasive. Experts recommend screening with this method every five years. However, if abnormalities such as polyps are detected, a colonoscopy is required for removal [49,58].

### 4.4. Liquid Biopsy

Liquid biopsies are also a promising emerging approach in colorectal cancer, analyzing DNA, cells, and other tumor-derived materials in body fluids such as blood and urine. This technique is currently being studied to quantify therapy responses, diagnose treatment resistance, and enable the early detection of colorectal cancer [59]. Tumor-educated blood platelets (TEPs) are another emerging promising biomarker due to their tumor-interacting RNA profiles [60]. TIMP1 mRNA, identified through RNA sequencing, is significantly elevated in platelets from colorectal cancer patients compared to healthy controls and patients with inflammatory bowel disease. TIMP1 mRNA promotes tumor growth by transferring into colorectal cancer cells, highlighting its potential as an independent diagnostic biomarker and its role in tumor progression [41]. Circulating extracellular vesicles (EVs) may have a role in cancer detection as they carry DNA, RNA, and proteins, reflecting tumor biology. Glypican-1 (GPC1)-positive EVs show high sensitivity (up to 97.3%) in GI cancer detection [61].

## 5. Pancreatic Cancer

Pancreatic cancer is a leading cause of cancer-related mortality worldwide. Due to the lack of specific signs, clinical symptoms, and screening protocols, it is often diagnosed in the late stages. Hence, testing for the early detection of pancreatic cancer is being investigated and remains an area of active research.

### 5.1. Liquid Biopsy

Liquid biopsies are minimally invasive blood tests used to detect ctDNA, CTCs, and other biomarkers released into the blood from tumors. Detection methods such as next-generation sequencing (NGS) have provided high sensitivity, enabling the identification of low-frequency mutations in ctDNA [62]. Recently, methylated cell-free DNA (cfDNA) derived from liquid biopsies has shown promise as a novel biomarker, demonstrating superior discriminatory power compared to traditional markers such as CA19-9 for distinguishing pancreatic cancers from non-cancerous conditions, including pancreatitis [42]. In pancreatic cancer, ctDNA is utilized as an essential tool for diagnosis, monitoring, and prognosis.

KRAS mutations are present in approximately 63–93% of pancreatic ductal adenocarcinoma (PDAC) cases and represent a key driver mutation. The detection of KRAS mutations in ctDNA holds promise for early diagnosis and disease monitoring. Studies suggest that KRAS mutations in ctDNA can be detected before imaging abnormalities are apparent, providing an opportunity for earlier intervention [63]. The presence of mutant KRAS ctDNA is associated with poor prognosis, as patients with detectable KRAS mutations in plasma have significantly shorter survival times compared to those without detectable mutations [63,64]. Moreover, ctDNA dynamics, particularly changes in mutant KRAS levels, have been shown to reflect treatment response more reliably than traditional markers such as CA19-9, with rapid increases in ctDNA levels often predicting disease progression and shorter survival [65].

Other key biomarkers include TP53, CDKN2A, and SMAD4 mutations. TP53 mutations, found in 50–75% of pancreatic cancers, prevent DNA damage recognition, disrupting cell cycle checkpoints and enabling tumor progression [62]. When detected alongside KRAS mutations in pancreatic juice samples using advanced sequencing techniques, TP53 mutations offer high diagnostic specificity [66,67]. CDKN2A mutations, occurring in approximately 98% of sporadic pancreatic cancers, lead to aggressive tumor behavior and poorer prognosis by disrupting the G1/S checkpoint [67]. SMAD4 mutations, present in about 55% of pancreatic cancers, impair TGF-β signaling, promoting tumor invasion and metastasis. These mutations are predominantly found in the advanced stages, making them valuable for identifying aggressive cancers [67].

Protein biomarkers also play an important role in pancreatic cancer diagnostics. CA 19-9, an FDA-approved biomarker, has limitations in sensitivity for early-stage detection and is affected by factors such as biliary obstruction. Combining CA 19-9 with other markers, such as carcinoembryonic antigen (CEA), improves diagnostic accuracy. Glypican-1 (GPC1), a membrane-anchored protein found in exosomes, has demonstrated near-perfect diagnostic accuracy in early studies, with 100% sensitivity and specificity in some cohorts [43].

Exosomal biomarkers, including RNA, proteins, and DNA, offer additional diagnostic potential by reflecting the molecular landscape of pancreatic cancer. GPC1-positive exosomes are associated with advanced-stage disease and shorter survival, showing superior sensitivity and specificity compared to CA 19-9 [44]. Exosomal microRNAs (miRNAs) such as miR-10b and miR-30c have demonstrated diagnostic potentials that often surpass traditional serum biomarkers [62]. Furthermore, KRAS and TP53 mutations detected in exosomal DNA show promise for the early diagnosis and monitoring of treatment responses [62].

### 5.2. VOCs in Breath Analysis

VOCs are being studied for the early detection of pancreatic cancer by reflecting tumor-induced metabolic changes. This emerging diagnostic tool has significant potential to enhance early diagnosis.

Pancreatic cancer disrupts metabolic pathways, leading to the production of specific VOC profiles, such as aldehydes, alcohols, and hydrocarbons. The dysregulation of enzymes such as aldehyde dehydrogenase (ALDH1) contributes to these metabolic shifts, influencing the levels of compounds such as benzaldehyde and acetone [68].

Advanced technologies are driving this field forward. Gas chromatography–mass spectrometry (GC–MS) has identified key VOCs associated with pancreatic cancer, demonstrating strong potential in distinguishing cancer cases from non-cancer cases [68]. Additionally, the ReCIVA™ Breath Collection System ensures reproducible and reliable alveolar breath sampling, maintaining VOC stability during analysis and proving effective in multi-center studies [68].

### 5.3. Advances in Imaging with Artificial Intelligence (AI)

Advanced imaging technologies, combined with AI, are transforming pancreatic cancer diagnostics by improving precision, enabling early detection, and enhancing staging accuracy. AI-enhanced MRI and CT scans have proven effective in identifying subtle early-stage pancreatic tumors, including lesions smaller than 2 cm, with sensitivities reaching 89.7% [69]. These tools also improve diagnostic accuracy by quantifying tumor size, vascular involvement, and textural changes, which are critical for staging and treatment planning [70]. Additionally, fast region-based convolutional neural networks (R-CNNs) process CT and MRI images within seconds, surpassing manual analyses by radiologists in both speed and precision [71]. Radiomics further enhances diagnostics by extracting high-dimensional features such as shape, texture, and intensity, which provide insights into tumor aggressiveness and therapy response [71]. For instance, radiomic analysis of diffusion-weighted MRI data has shown 87% sensitivity in predicting survival rates in patients with pancreatic ductal adenocarcinoma (PDAC) [71]. Integration of AI-driven imaging with other diagnostic tools, such as liquid biopsies detecting ctDNA and KRAS mutations or VOC profiling, further improves diagnostic precision by linking molecular and imaging findings [71]. AI also facilitates the early detection of precancerous lesions, such as intraductal papillary mucinous neoplasms (IPMNs), which are often challenging to diagnose with imaging alone [72,73].

### 5.4. Multi-Omics Integration

This approach combines data from genomics, transcriptomics, proteomics, and metabolomics to create a detailed profile of pancreatic cancer biomarkers, paving the way for earlier detection and more personalized treatment strategies [74,75]. Genomics focuses on identifying mutations in key genes such as KRAS, TP53, CDKN2A, and SMAD4 [76]. Transcriptomics examines RNA transcripts to reveal patterns of gene expression and regulatory networks. Proteomics measures proteins, such as glypican-1 (GPC1), to gain insights into tumor function and potential diagnostic markers. Metabolomics analyzes metabolic changes, including alterations in glucose and lipid metabolism, which are commonly associated with pancreatic cancer. By combining these layers of information, multi-omics provides a holistic understanding of tumor biology, enabling more targeted and effective interventions [77].

### 5.5. Future Innovations

Future directions and innovations in diagnostics include the use of patient-derived organoids to test and refine biomarkers for liquid biopsies and VOC analysis. Nanotechnology is advancing the development of nanoparticle-based platforms for capturing and analyzing ctDNA or exosomal biomarkers with enhanced precision [78]. Combining liquid biopsy, VOC analysis, and AI imaging into a multimodal diagnostic pipeline shows promise for maximizing early detection rates [79]. Additionally, portable point-of-care devices integrating VOC detection or ctDNA analysis could facilitate diagnostics in community settings [80]. Table 1 summarizes the diagnostic performance of prominent non-invasive biomarkers across gastrointestinal cancers, highlighting their potential roles in early detection and prognosis.

## 6. Hepatocellular Carcinoma

Hepatocellular carcinoma (HCC) is a leading cause of cancer mortality globally and ranks as the sixth most common cancer worldwide. Most cases of HCC are associated with chronic liver diseases and cirrhosis, often caused by metabolic-associated steatotic liver disease (MASLD)/metabolic-associated steatohepatitis (MASH), alcohol-related cirrhosis, hepatitis B, and hepatitis C. According to AASLD guidelines, patients with cirrhosis should undergo HCC surveillance with liver ultrasound and serum alpha-fetoprotein (AFP) every six months. However, there are no established guidelines for surveillance in patients with advanced fibrosis. Most patients with HCC are asymptomatic in the early stages, leading to delays in diagnosis and high mortality rates. Early detection through effective surveillance strategies for at-risk populations is critical for improving patient outcomes and survival rates [81].

### 6.1. Biomarkers

The screening and early detection of HCC remain challenging due to its asymptomatic nature in the early stages and its occurrence in patients without cirrhosis [82]. A thorough clinical history, high-quality imaging, and reliable biomarkers are essential for early diagnosis. An ideal biomarker should be specific, sensitive, reproducible, cost-effective, and capable of correlating with tumor stages while providing rapid results. Serum AFP is a traditional biomarker for HCC but has limitations due to low sensitivity and specificity, as levels may be elevated in non-HCC conditions or remain normal in the presence of HCC. Therefore, AFP is not sufficient as a surveillance tool by itself and is often combined with imaging. Other protein biomarkers include des-γ-carboxy prothrombin (DCP) and AFP-L3, which can be used alongside AFP. DCP has demonstrated better sensitivity than AFP and has shown efficacy in detecting HCC and predicting its radiological and histological characteristics. AFP-L3, a subtype of AFP found in cancer cells, provides diagnostic specificity by measuring its proportion to total AFP (AFP-L3%) [83].

### 6.2. Liquid Biopsy

ctDNA can reveal genetic and epigenetic changes associated with cancer through a simple blood sample. The analysis of ctDNA methylation patterns shows promise for HCC detection. Panels such as the HelioLiver Test, Oncoguard Liver Test, and GALAD score have been developed using ctDNA analysis. The GALAD score, validated in the USA, incorporates gender, age, and levels of AFP, AFP-L3, and DCP, showing high accuracy for HCC detection, particularly in cirrhotic patients. The HelioLiver Test combines circulating cell-free DNA methylation markers with demographic data and tumor markers, while the Oncoguard Liver Test incorporates methylation biomarkers such as HOXA1, TSPYL5, and B3GALT6 along with AFP [45].

### 6.3. Non-Coding RNA Biomarkers

Emerging RNA biomarkers, including miRNAs, long non-coding RNAs (lncRNAs), and circRNAs, also hold potential for HCC diagnosis. Circulating miRNAs, such as miR-122, miR-21, and miR-221, exhibit distinct expression patterns and have demonstrated diagnostic accuracy comparable to traditional biomarkers. Circular RNAs have shown promise in HCC diagnosis [84]. Urine and stool sample miRNAs offer an additional avenue for developing diagnostic and therapeutic markers, representing a promising area for advancement in HCC detection.

### 6.4. Metabolomics

Metabolomics, which involves detecting and characterizing small metabolites, also provides valuable information about cellular metabolic changes in HCC. Potential markers include bile, phospholipids, peptides, sphingolipids, amino acids, and modified nucleosides such as 1-methyladenosine (M1A) [85]. These metabolomic changes offer potential for the early detection of HCC in the future. Extracellular vehicles (EVs), which are membrane vesicles involved in intracellular communication, contain proteins that can serve as diagnostic and therapeutic markers for HCC. EVs can enhance drug delivery accuracy and exhibit direct antitumor effects, though their extraction remains costly and further research is required to fully explore their potential [85].

### 6.5. Radiomics

Radiomics also plays a role in HCC surveillance, with ultrasound being a widely used, non-invasive tool. However, ultrasound is limited by operator dependency. AI models, such as convolutional neural networks (CNNs), improve diagnostic accuracy by reducing variability and optimizing data analysis. AI-based models have shown specificity and sensitivity rates of 81–97% in detecting and classifying focal liver lesions. Advanced AI systems, such as YOLO (You Only Look Once) models, offer superior real-time detection and have extended capabilities in diagnosing malignant liver lesions, cholangiocarcinoma, and regenerative liver nodules. Despite their promise, external validation and real-time application remain necessary [86].

### 6.6. Combination Approach

Lastly, a multi-omics approach integrating genomics, transcriptomics, proteomics, glycomics, and metabolomics offers a deep vision into the molecular mechanisms of HCC development, enabling earlier and more accurate detection, as well as developing better treatments for HCC. However, the heterogeneity and complexity of HCC pose significant challenges, highlighting the need for further research to standardize surveillance strategies for diverse at-risk populations [87].

## 7. Small Bowel Cancers

Small bowel cancers, although rare, present a big challenge for diagnosis because of anatomic location and non-specific symptoms. There are emerging non-invasive screening methods, in particular liquid biopsies, for improving early diagnosis and management. The following approaches outline the developments in this respect.

### 7.1. Liquid Biopsy

Liquid biopsies enable early detection and personalized treatment by identifying tumor-derived biomarkers in circulation. Mutations in KIT, PDGFRA, and APC are commonly linked to small bowel tumors, including adenocarcinomas, neuroendocrine tumors (NETs), and gastrointestinal stromal tumors GISTs [88]. CTCs provide molecular insights for disease monitoring. Advanced microfluidic and immunomagnetic separation techniques achieve 85.3% sensitivity and 90.3% specificity for detecting CTCs in gastrointestinal cancers [46]. Recent advancements in plasma protein-based diagnostics enhance accuracy in NET detection. The EXPLAIN study used supervised machine learning to differentiate pancreatic neuroendocrine tumors (PanNETs) from small intestinal neuroendocrine tumors (SI-NETs). The PanNET model achieved 84% sensitivity, 98% specificity, and an AUROC of 0.99, while the PanNET vs. SI-NET model reached an AUROC of 0.98, highlighting the potential of multi-protein strategies for improving NET diagnostics [89]. Additionally, exosomal biomarkers such as glypican-1 (GPC1) show 97.3% sensitivity in pancreatic cancers, with potential applications in small intestinal malignancies [47]. Liquid biopsy advancements continue to refine non-invasive cancer detection and monitoring.

### 7.2. Emerging Approaches

Epigenetic markers, such as promoter methylation and RNA-based biomarkers, have promises for the early detection of small bowel cancers. DNA hypermethylation in tumor suppressor genes such as CDO1, RASSF1, and SFRP1 is a hallmark of early-stage gastrointestinal cancers. These highly relevant methylation genes (or HRMGs) have demonstrated a sensitivity and specificity above 90% in distinguishing cancerous versus normal tissues [90]. Biomarkers based on RNA also have great importance in this regard. Overexpression of miRNAs, including miR-21 and miR-155, was demonstrated in gastrointestinal malignancies, including small bowel malignancies, thus offering potential biomarkers for early diagnosis [90]. Differential expression of some lncRNAs, including HOTAIR and MALAT1, has further been associated with tumor progression and poor prognosis; thus, they have possible applications in liquid biopsy [90]. A comparison of emerging non-invasive screening techniques is provided in Table 2.

### 7.3. Complementary Screening Techniques

Advancements in AI have significantly improved the detection and diagnosis of small bowel diseases. CE, supported by AI algorithms, has revolutionized the visualization of the small intestine. AI models such as CNNs have demonstrated superior accuracy in segmenting the small bowel, enabling enhanced preoperative planning and disease evaluation [91], AI-supported CE systems reduce video review times while maintaining diagnostic precision, assisting clinicians in detecting subtle mucosal changes [91], leading to the early identification of pathological features, including small intestinal tumors, with high sensitivity and specificity. For example, a CNN-based system achieved 98.7% sensitivity and 96.6% specificity in diagnosing primary small intestinal tumors from CE images [91].

## 8. Limitations

While this review provides a comprehensive overview of major gastrointestinal (GI) cancers, including esophageal, gastric, colorectal, pancreatic, hepatocellular, and small bowel cancers, it does not extensively cover other GI malignancies such as bile duct cancer (cholangiocarcinoma), gallbladder cancer, and cancer of the ampulla of Vater. These cancers, although less common, have distinct pathogenesis, diagnostic challenges, and management approaches that warrant separate in-depth discussions. Future reviews should aim to address these malignancies to provide a more holistic perspective on GI cancer detection and management.

## 9. Conclusions, Challenges, and Considerations

While the non-invasive screening of gastrointestinal malignancies provides important steps toward the goal of early diagnosis, several challenges remain to be resolved regarding their sensitivity, accessibility, and routines in clinical practice. First, there is a variation in sensitivity and specificity regarding different tests and types of cancers. Whereas some biomarkers are very accurate, others may yield false positives, with all the consequences of superfluous invasive procedures, or, on the other hand, false negatives, which can delay the diagnosis. The presence of such genetic, environmental, and lifestyle heterogeneity therefore makes it difficult to have common biomarkers for gastrointestinal cancers that will result in similar predictive values in different populations. Large-scale multi-center biomarker panel standardization and validation studies are extremely important to achieve reliability and clinical utility. Cost-effectiveness is another big issue, specifically in developing countries where not every facility may possess advanced molecular and AI-driven diagnostic capabilities. High costs associated with next-generation sequencing, liquid biopsies, and AI-enhanced imaging could dampen the likelihood of widespread adoption and thus increase disparities in healthcare.

Regulatory challenges also continue to play an important role, with many of these emerging technologies still awaiting full approval from governing bodies such as the FDA, EMA, and others. Longitudinal studies with longer follow-ups will be required to determine clinical benefits, establish refinement of screening intervals, and allow incorporation into existing guidelines. The adoption of non-invasive screening will also require engagement between clinicians, researchers, and policymakers in terms of the most appropriate clinical pathways and follow-up for individuals with positive tests. Another crucial issue is compliance and education at the patient level.

**Figure 3:**
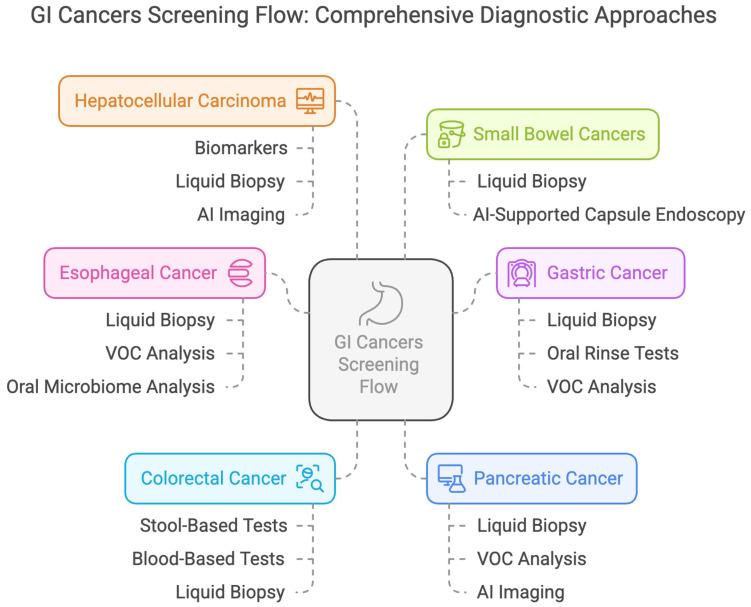
Comprehensive Screening Flow for Gastrointestinal Cancers.

Non-invasive screening methods are designed to improve adherence compared to traditional invasive procedures such as endoscopy and colonoscopy (Figure 3). However, awareness, trust, and understanding of these novel technologies by patients are major factors that will determine the uptake and use of these technologies. Test accuracy, benefits, and potential risks need to be communicated to encourage participation, especially in high-risk populations who may already face barriers to screening. Of importance are ethical considerations that also must be considered concerning genetic and epigenetic testing, data privacy, incidental findings, and the psychological consequences for the patient with risk stratification. Moreover, integrating multi-omics approaches with AI-driven diagnostics raises questions about data security and a need for robust computational infrastructures that can manage, analyze, and interpret big biological and imaging data.

It also involves the challenge of standardizing AI algorithms across diverse health systems, ensuring non-bias in training datasets and eliminating algorithmic biases. Furthermore, as promising as AI-enhanced imaging and molecular diagnostics are, they should supplement and not supplant human clinical skills and be based on structured training for health providers that enables their effective use. Ultimately, such challenges require a multidisciplinary approach involving clinicians, researchers, regulatory bodies, and policymakers. Further investment in large-scale clinical trials, technology, and healthcare infrastructure is required to refine the non-invasive screening methods and make them more sensitive, available, and affordable. Only then can these innovations revolutionize early gastrointestinal cancer detection and improve patient outcomes, reducing the global burden of GI malignancies.

## Figures and Tables

**Table 1:** Sensitivity, Specificity, and Diagnostic Potential of Non-Invasive Biomarkers in GI Cancers.

Cancer Type	Biomarker/Method	Sensitivity (%)	Specificity (%)	Diagnostic Utility	Reference
Esophageal Cancer (EC)	Circulating miRNAs	80	81	Early detection of ESCC and EAC	[11]
Esophageal Cancer (EC)	5-MDM Panel (Methylated DNA Markers)	74 (EAC), 78 (ESCC)	92	High accuracy for esophageal cancer detection	[13]
Esophageal Cancer (EC)	VOC Breath Analysis (5-VOC model)	94.1	96	Non-invasive breath test for EC detection	[18,19]
Gastric Cancer (GC)	CancerSEEK (ctDNA + proteins)	70	>99	Multianalyte blood test for GC diagnosis	[29]
Gastric Cancer (GC)	Oral Microbiome Profiling	85 (AUC 0.87)	-	Distinguishes GC and pre-malignant lesions	[33]
Colorectal Cancer (CRC)	Shield (Blood-based test)	83	90	Plasma DNA-based screening for CRC	[39]
Colorectal Cancer (CRC)	Cologuard (mt-sDNA test)	92	87	Stool-based multi-target DNA test	[40]
Colorectal Cancer (CRC)	TIMP1 mRNA (Tumor-Educated Platelets)	95.8 (95% CI)	-	Emerging platelet-based biomarker for CRC	[41]
Pancreatic Cancer	Methylated cfDNA (Liquid Biopsy)	85	88	Detects pancreatic cancers with superior accuracy	[42]
Pancreatic Cancer	GPC1-Positive Exosomes	100	100	Early-stage detection with near-perfect accuracy	[43,44]
Hepatocellular Carcinoma	GALAD Score	High (exact not specified)	High	Integrates biomarkers and demographics for HCC	[45]
Small Bowel Cancers	CTC Detection (Microfluidic)	85.3	90.3	Liquid biopsy for small bowel tumor detection	[46]
Small Bowel Cancers	Exosomal Biomarkers (GPC1)	97.3	-	Potential application in small intestinal malignancies	[47]

**Table 2:** Comparison of Non-Invasive Screening Techniques for Major GI Cancers.

Screening Method	Applicable Cancer(s)	Advantages	Limitations	Recommended Interval
Liquid Biopsy (ctDNA, cfDNA, CTCs)	Pancreatic, CRC, EC, HCC	Minimally invasive; dynamic tumor monitoring	Low sensitivity in early stages; cost-intensive	Ongoing research; not routine
Stool DNA Testing (mt-sDNA)	Colorectal Cancer (CRC)	High sensitivity; detects non-bleeding lesions	High cost; follow-up colonoscopy for positives	Every 3 years
VOC Breath Analysis	Esophageal, Gastric, Pancreatic	Non-invasive; rapid results	Requires further validation; differentiation issues	Under investigation
Oral Microbiome Analysis	Gastric, Esophageal Cancer	Easy sample collection; potential early detection	Affected by lifestyle factors; requires standardization	Research stage
Capsule Endoscopy	Small Bowel, Esophageal	Non-invasive; good visualization	No biopsy retrieval; labor-intensive interpretation	Every 5 years (if applicable)
AI-Enhanced Imaging (CT, MRI, CE)	Pancreatic, HCC, Small Bowel	Improves diagnostic accuracy; early tumor detection	Requires computational infrastructure; costly	Case-specific
Exosomal Biomarkers (miRNA, GPC1)	Pancreatic, CRC, HCC	High sensitivity and specificity; dynamic monitoring	Technical complexity; expensive	Ongoing research
Fecal Methylation Testing	Colorectal Cancer (CRC)	High specificity; detects DNA changes independent of bleeding	Limited availability; validation needed	Every 3 years
AI-Driven Radiomics	Pancreatic, HCC	Predicts tumor aggressiveness; non-invasive	Requires large datasets for validation	Research stage

## Data Availability

All data utilized for this narrative review are publicly available on PubMed and embedded in the reference section of the manuscript.
